# Supplementary material for: An integrated continuity of care measure improves performance in models predicting medication adherence using population-based administrative data
Source: PLoS One. 2022 Mar 3;17(3):e0264170. doi: 10.1371/journal.pone.0264170 (PMC8893672; doi:10.1371/journal.pone.0264170)
Supplement: Attachment — Submitted filename: Response to Reviewers.docx [file pone.0264170.s001.docx]

Dear Dr. Liu,

We thank the Editors and reviewers for the careful review of our paper. We have responded to each point raised and made the requested changes in the manuscript. We believe these changes have strengthened the article.

We have uploaded a revised version with changes marked along with a clean version.

We look forward to hearing from you.

Yours sincerely,

Shenzhen Yao, PhD and David Blackburn PharmD

**Reviewer #1:**

Thanks very much for this opportunity to review this interesting study. This study aimed to improve the measure of COC, based on a more comprehensive perspective, and use it to predict medication adherence. The results comparing the original measures of COC and this new-developed measure also suggested its advantage on predicting medication adherence and give some implications to clinical practices. Here are some concerns raised for authors to consider.

Background

1. The introduction of the current study seemed insufficient, especially some basic background information missed. Considering the aim of the current study is to improve the medication adherence, the prevalence of non-adherence and its consequences should be added to highlight the significance of the current study. In addition, medication adherence is a complex process and authors may need to briefly summary it and further swift the focus on the effect of COC.

**RESPONSE:** We agreed with the reviewer, and have added a new paragraph at the very beginning of the paper to provide more context about non-adherence in general. We have also reworded other parts of the Introduction section to address the non-adherence issue.

2. The introduction mentioning the mediation effect of patient-physician relationship (1st paragraph) seemed useless based on the aim and design of the current study. Why authors have to mentioned this effect if this kind of factors were not considered/adjusted in the current study?

**RESPONSE:** Our intention was simply to outline the theoretical pathway that links COC with adherence. We have made this the second paragraph of the paper now (with a general paragraph on adherence to start). We hope it makes more sense now.

Methods
3. (Data sources) It seemed the median household income quintiles is estimated by linking the first three digits of postal code to Statistics Canada Census data. Pls provide a further interpretation for the validity of this method and relevant references.

**RESPONSE:** A reference is now added following the sentence “…linking the first three digits of postal code to Statistics Canada Census data…” in the first paragraph of the section “Data sources”. The reference is to indicate that the method of linking to census data by first three digits of postal code is a common practice in Canada, and is endorsed by the Canadian Institute for Health Information (CIHI). We have also added a follow-up sentence highlighting the known limitations of area-based income estimation.

4. (Study design and population) The cohort period seemed from 2012-2017, while this study tried to identify new initiation as receiving no dispensations for a statin medication in the five years prior. I noticed that the data ranges from 1999. It seemed confused why authors choose this period for construction of the current cohort rather than the overall period of data available (for example, 2004-2017 to ensure 5 years prior dispensing data). Is there any specific reason?

**RESPONSE:** We included new statin users between 2012 and 2017. To determine a new statin user, we looked back for 5 years to ensure that the user did not receive a statin medication. However, we used a longer window for identifying chronic conditions [i.e., back to 1996 (not 1999)] for inclusion in our multivariable analyses to adjust for potential confounding in case the presence of chronic diseases were recorded prior to the beginning of statin therapy.

5. (Study design and population) The reasons why statin medications were selected in the current study may be insufficient. Though some aspects were mentioned by authors, however, why these aspects may influence the selection of medication should be further interpreted (as well as references).

**RESPONSE:** We have added greater detail on the utility of statin medications as a model for adherence in general (1^st^ paragraph under “Study Design and Population”).

6. The number of exclusion criteria is disordered, pls re-number them.

**RESPONSE:** Corrected.

Results
7. (Table 3) **mis-writing of “Among patients with high UPCIf**”

**RESPONSE:** In our submitted word document, it is ‘UPCI^f^’ – the last letter being the mark of a footnote. The formatting might get lost during transformation of the document.

Discussion
8. Considering the main contribution of the current study focused on the newly-developed measure of COC and its application. It would be necessary for authors to further discuss its implication for clinics and future research. Is the comprehensive measure possible for further use to improve COC, what is the advantage as well as potential unintended consequences of this COC measures compared with existing UPCI and etc.

**RESPONSE:** We agree with the reviewer on that the integrated COC should be extensively discussed. As such, we now have added the following paragraph in the discussion section:

“Population-based models of non-adherence generally have incomplete covariate adjustment and strategies are needed to capture clinical (e.g., side effects) and behavioural measures (e.g., attitudes and beliefs) .[2] However, our study demonstrated that improving on the measurement of existing variables may also help account for unexplained variance. The success of our integrated COC measure was likely due to the inclusion of diverse clinical services from a single practitioner rather than relying solely on the number of physician visits. However, it remains unknown whether the integrated COC measure could be improved further. Despite the identification of CMEP as a marker of COC in previous studies,[17] it is possible that other service claim codes could replace (or be added to) CMEP. If complete medical examination claims were not necessary for optimal COC, then the contribution of this criterion was likely as a marker of diverse clinical service provision rather than a specific effect of CMEP itself. This issue is important to evaluate because the utilization and utility of CMEP may vary significantly depending on the jurisdiction/health system. We are hopeful our research will stimulate further work in the area and provide more information about the potential for health administrative databases to identify service patterns associated with primary care practices that promote optimal medication adherence.”

**Reviewer #2:**

Overall, this paper is clear and presents a well-executed study on continuity of care (COC) measures and their relationship to medication adherence. The case is well-made for the value of a COC measure that integrates usual statin provider and complete medical examination provider with the conventional usual care provider concept.
I have several specific suggestions for improving the manuscript:

1. More specific title. The paper is really about the value of an integrated COC measure. I think that should be in the title.

**RESPONSE:** We agreed with the reviewer and the title is now changed to: “An integrated continuity of care measure improves performance in models predicting medication adherence using population-based administrative data.”

2. It’d be good if it were easier for the reader to see that ‘integrated COC measure’ means integrating usual statin and complete medical examination provider with usual GP – ideally in the abstract, and I think also in the conclusion. The phrasing from the last paragraph of the Introduction, “integrated COC measure consisting of physician visits, prescribing, and claims for a complete medical examination” is a good example of language that clarifies what the integrated COC measure is, but I feel it should be more prominent given that it’s essentially the subject of the paper.

**RESPONSE:** We agreed with the reviewer and the last sentence in the Objectives of the Abstract is now modified as (adding the parts in bold font). We also added the components of the integrated COC measure in the conclusion of the paper.

“This study aimed to: a) determine if high UPCI values predict physicians who deliver different clinical services; and b) compare UPCI with an integrated COC measure **requiring evidence for** **physician visits, prescribing, and a complete medical examination** in a multivariable model of patients receiving statin medications.”

3. Results Fig 1 – cohort flowchart is missing in the manuscript I reviewed.

**RESPONSE:** We uploaded fig1 as a separate file as instructed in the journal’s website. We hope that the reviewer can view that separate file.

4. Evidently a prescriber ID is in the dispense record (prescription drug claims file), but this is not stated – see last sentence of Data Sources.

**RESPONSE:** The sentence is now revised as (adding the part in bold font):

“Each claim includes a Health Canada drug identification number (DIN), a dispensation date, the quantity dispensed, total cost (including medication acquisition cost and markup/dispensing fee), **the encrypted identification code of the prescriber (the same physician as in the physician service claims file)**, and the proportion covered by government insurance”

5. The word ‘included’ is used repeatedly in Covariates (and also in footnote g of table 3) – are these all the covariates or just examples from a much longer list? If the list is complete please say ‘were’ (e.g. ‘The patient covariates were age, sex, and residence (rural/urban) on the index date’). Perhaps include an appendix or supplementary material if the covariates in the adjusted model are too extensive to list in the body of the paper.

**RESPONSE:** The sentences were changed using ‘were’ instead of ‘included’.

6. Results: The authors say 60.1% had no complete medical exam or received it from a specialist – can we have a breakdown between those alternatives?

**RESPONSE:** The break-down is now provided and the sentence revised as (adding the part in bold font):

“The rest 33,127 (60.1%) of the patients either had no complete medical examinations during the follow-up period **(322,41, 58.5% of 55,144)** or received the examinations from a specialist **(886, 1.6% of 55,144)**.”

7. Not enough is made of the sensitivity of predictor performance to the threshold used in defining ‘high UPCI’. The median for UPCI is reported as a high-sounding 0.82, whereas integrated COC is rarer at 28.3% - is this why it’s a better predictor of adherence? Apparently not entirely… it’s great that you’ve included the better performance with 25th percentile UPCI – OR 1.39 v 1.56 instead of 1.23 v 1.56. (Incidentally this seems consistent with lowest 33% of UPCI being distinct from middle and highest 33% for adherence in your reference [4].) That said, you didn’t go back to see how this more effective UPCI cut-off performed for predicting enhanced COC, or usual statin or complete medical exam provider.

**RESPONSE:** We agreed with the reviewer. Indeed we compared the predictive performance of integrated COC vs UPCI at 25^th^/75^th^ percentile using DeLong test. The results showed that the model with integrated COC was always significantly improved. We have now included these results in the manuscript (in the last paragraph of the Results section):

“The DeLong test showed that integrated COC term significantly improved the AUROC compared to the models using different thresholds of the UPCI measure (+0.004, χ2 statistic = 16.0, p < 0.0001 when threshold was set at the 25th percentile of UPCI; +0.009, χ2 statistic = 16.0, p < 0.0001 when threshold was set at the 75th percentile).”

8. As a more general comment on the point above, the use of data from one particular healthcare system should be cited as a limitation. You have shown that it’s possible to formulate an effective integrated COC measure quite elegantly in the Saskatchewan health system. But this measure is surely sensitive to the rates at which complete medical examinations are performed as well as the median (and distribution) of UPCI. I’m not an authority on this, but I take it that comprehensive annual medical checks are normal in, say, Japan; and I presume they’re much rarer in some other jurisdictions. Similarly, the role of the GP is strong in much of the commonwealth, but may not extend to regular statin provision in other systems. At any rate, please note the use of data from one system as a limitation, and consider further discussion of how the model may or may not apply more broadly.

**RESPONSE:** The role of comprehensive medical examinations in measure of COC is now further discussion in the following sentences added in the discussion section:

“…If complete medical examination claims were not necessary for optimal COC, then the contribution of this criterion was likely as a marker of diverse clinical service provision rather than a specific effect of CMEP itself. This issue is important to evaluate because the utilization and utility of CMEP may vary significantly depending on the jurisdiction/health system. We are hopeful our research will stimulate further work in the area and provide more information about the potential for health administrative databases to identify service patterns associated with primary care practices that promote optimal medication adherence.”

9. Typo: Last paragraph of Discussion, ‘we improved up on’ -> ‘we improved upon’

**RESPONSE:**

Changed to ‘we improved upon’
