# Supplementary material for: An integrated continuity of care measure improves performance in models predicting medication adherence using population-based administrative data
Source: PLoS One. 2022 Mar 3;17(3):e0264170. doi: 10.1371/journal.pone.0264170 (PMC8893672; doi:10.1371/journal.pone.0264170)
Supplement: Attachment — Submitted filename: Response to reviewers v20220119.docx [file pone.0264170.s002.docx]

Dear Dr. Liu,

We thank the Editors and reviewers for the careful review of our paper. We have responded to each point raised and made the requested changes in the manuscript. We believe these changes have strengthened the article.

We have uploaded a revised version with changes marked along with a clean version.

We look forward to hearing from you.

Yours sincerely,

Shenzhen Yao, PhD and David Blackburn PharmD

PONE-D-21-07353R1

An integrated continuity of care measure improves performance in models predicting medication adherence using population-based administrative data

Please include the following items when submitting your revised manuscript:

- A rebuttal letter that responds to each point raised by the academic editor and reviewer(s). You should upload this letter as a separate file labeled 'Response to Reviewers'.

**Authors’ response: included**

- A marked-up copy of your manuscript that highlights changes made to the original version. You should upload this as a separate file labeled 'Revised Manuscript with Track Changes'.

**Authors’ response: included**

- An unmarked version of your revised paper without tracked changes. You should upload this as a separate file labeled 'Manuscript'.

**Authors’ response: included**

If you would like to make changes to your financial disclosure, please include your updated statement in your cover letter. Guidelines for resubmitting your figure files are available below the reviewer comments at the end of this letter.

**Authors’ response: no changes**

If applicable, we recommend that you deposit your laboratory protocols in protocols.io to enhance the reproducibility of your results. Protocols.io assigns your protocol its own identifier (DOI) so that it can be cited independently in the future. For instructions see: <https://journals.plos.org/plosone/s/submission-guidelines#loc-laboratory-protocols>. Additionally, PLOS ONE offers an option for publishing peer-reviewed Lab Protocol articles, which describe protocols hosted on protocols.io. Read more information on sharing protocols at <https://plos.org/protocols?utm_medium=editorial-email&utm_source=authorletters&utm_campaign=protocols>.

**Authors’ response: not applicable**

**Review Comments to the Authors**

**Reviewer #1**: Thanks very much for this opportunity to review the revised draft of the current study. It’s much clear this time. There were only a few minor concerns for authors as follows.

1. The presentation of data sources could be improved. Authors may use subsections to make the presentation clearer (based on 5 files) or add a table to summarize this information.

**Authors’ response:** The data sources are now arranged in subsections.

2. Discussion, 1st paragraph, “Our results indicate that a high UPCI value did not identify physicians who were providing core services”. It seemed that the statement is not accurate. First, the current study showed that the UPCI index was partly able to predict other indicators (table 2); Second, it seemed that defining prescribing and examination as core services is too arbitrary. Thus, the statement may be revised to accurately reflect the improvement of integrated COC compared with UPCI.

**Authors’ response: revised. The sentence is now revised as:**

**“Our study was conducted exclusively on new statin users, yet the specific statin prescriber did not generate a high UPCI value in approximately half of the patients studied. However, adding statin prescribing and complete medical examination activities to the COC definition (i.e., with high UPCI value) resulted in a stronger association with medication adherence and significantly improved the predictive power of a medication adherence model.”**

**Reviewer #2:** The revisions are entirely to my satisfaction in addressing prior concerns. I'm particularly happy with the changes to the title, abstract and discussion.

One very minor point with the new sensitivity analysis text: "The DeLong test showed that integrated COC term significantly improved the AUROC compared to the models using different thresholds of the UPCI measure (+0.004, χ2 statistic = 16.0, p < 0.0001 when threshold was set at the 25th percentile of UPCI; +0.009, χ2 statistic = 16.0, p < 0.0001 when threshold was set at the 75th percentile)" I'm suspicious of both chi-squareds being 16.0 given the different changes in AUROC. Perhaps the 25th percentile one is 16.0 and the 75th percentile one is somewhat higher?? Just pointing out for the authors to check.

**Authors’ response: we thank reviewer pointing out the error. The sentence is now corrected (as highlighted) as:**

**“The DeLong test showed that integrated COC term significantly improved the AUROC compared to the models using different thresholds of the UPCI measure (+0.004, χ2 statistic = 16.0, p < 0.0001 when threshold was set at the 25th percentile of UPCI; +0.009, χ2 statistic = 83.1, p < 0.0001 when threshold was set at the 75th percentile).”**
